# Comparing Options for Deriving Chemical Ecotoxicity Hazard Values for the European Union Environmental Footprint, Part II

**DOI:** 10.1002/ieam.4169

**Published:** 2019-08-14

**Authors:** Erwan Saouter, Deidre Wolff, Fabrizio Biganzoli, Donald Versteeg

**Affiliations:** ^1^ European Commission, Joint Research Centre (JRC), Ispra Italy; ^2^ EcoStewardship, Cincinnati, Ohio USA

**Keywords:** REACH, Ecotoxicity, Environmental Footprint, SSD curve, CLP

## Abstract

Using the European Union's Registration, Evaluation, Authorisation and Restriction of Chemicals (REACH) ecotoxicity data, this paper compares 3 different approaches to calculate final substance toxicity hazard values using the USEtox approach (chronic EC50 + acute EC50/2), using only acute EC50 equivalent data (EC50_eq_), and using only chronic no observed effect concentration equivalent (NOEC_eq)_ data. About 4008, 4853, and 5560 substance hazard values could be calculated for the USEtox model, acute only, and chronic only approaches, respectively. The USEtox model provides hazard values similar to the ones based on acute EC50 data only. Although there is a large amount of variability in the ratios, the data support acute EC50_eq_ to chronic NOEC_eq_ ratios (calculated as geometric mean) of 10.64, 10.90, and 4.21 for fish, crustaceans, and algae respectively. Comparison of the calculated hazard values with the criteria used by the EU chemical Classification, Labelling, and Packaging regulation (CLP) shows the USEtox model underestimates the number of compounds categorized as very toxic to aquatic life and/or having long‐lasting effects. In contrast, use of the chronic NOEC data shows a good agreement with CLP. It is therefore proposed that chronic NOEC_eq_ are used to derive substance hazard values to be used in the EU Environmental Footprint. Due to poor data availability for some chemicals, the uncertainty of the final hazard values is expected to be high. *Integr Environ Assess Manag* 2019;15:796–807. © 2019 The Authors. *Integrated Environmental Assessment and Management* published by Wiley Periodicals, Inc. on behalf of Society of Environmental Toxicology & Chemistry (SETAC).

## Key Points

- The European Union (EU) Registration, Evaluation, Authorisation and Restriction of Chemicals (REACH) database has been used to calculate aquatic toxicity hazard values for 5560 chemicals substances to be used in the EU Environmental Footprint.
- Three methods to calculate hazard values have been compared using all available acute and chronic reference points.
- Hazard values derived from chronic no observed effect concentration equivalent (NOEC_eq_) showed the best agreement with the chemical toxicity ranking as defined within the EU chemical Classification, Labelling, and Packaging regulation (CLP) regulation.
- Acute EC50_eq_ to chronic NOEC_eq_ ratios of 10.64, 10.90, and 4.21 for fish, crustaceans, and algae, respectively, have been calculated.

## INTRODUCTION

In April 2013, the European Commission (EC) published a recommendation on the use of a common method to measure and communicate the life cycle environmental performance of products and organizations (EC 2013), the Environmental Footprint (EU‐EF). In November 2013, the EC started a 4‐y pilot exercise with industries, nongovernmental organizations (NGOs), and academia to test the application of the EU‐EF method on 25 products (e.g., batteries, paints, water supply pipes, detergents). The goal was to produce specific product category rules (PCRs) to help future applicants calculate the impact of their product in a standardized, efficient, and cost‐effective manner. The latest version of the guideline has been recently published by the EC (Zampori and Pant 2019). The EU‐EF considers 16 different impact categories: climate change; ozone depletion; freshwater ecotoxicity; human toxicity, cancer; human toxicity, noncancer; particulate matter; ionizing radiation; photochemical ozone formation; acidification; terrestrial eutrophication; freshwater eutrophication; marine eutrophication; resource depletion, water; resource depletion, mineral and metals; resource depletion, fossil; and land use. Experience gained and the recommendations to further improve the EU‐EF method have been summarized in various reports (Kerkhof etal. 2017; Vincent‐Sweet etal. 2017) and presentations (EC 2018a). One important decision made during the final EU‐EF development phase was to stall the implementation of the 3 chemical toxicity impact categories from the mandatory requirements until the underlying model for these impact categories is improved. The model initially recommended by the EC to calculate the impact of chemical was USEtox (Hauschild etal. 2008; Henderson etal. 2011; Fantke etal. 2017). The main criticisms voiced via the pilots were that model results were difficult to understand and interpret, leading to, for example, the identification of unexpected chemicals as main contributors to overall toxicity. Furthermore, the source of the input data including physicochemical parameters as well as ecotoxicity values were questioned, which led to the recommendation to rely on the recent chemical database resulting from the implementation of the EU Registration, Evaluation, Authorisation and Restriction of Chemicals (REACH) legislation (EC 2006). Following the EU‐EF pilots’ feedback, the EC Joint Research Centre (EC‐JRC) conducted a review of the USEtox model focusing on opportunities for improvements for use in the context of the EU‐EF method and exploring the possibility to use the REACH database to compile toxicity data on freshwater organisms (Saouter etal. 2017a, 2017b).

In February 2018, a workshop was organized with all EU‐EF stakeholders to review the finding concerning USEtox and agree on possible ways to calculate chemical ecotoxicity hazard values to be used in the EU‐EF. The main conclusions were that

1) toxicity should be preferably based on chronic toxicity test results making use of the endpoint available (mainly no observed effect concentration [NOEC], low observed effect concentration [LOEC], concentration causing 10 to 20% effect [EC10 to EC20]), and
2) ranking of the hazard values used in the EU‐EF should be logically aligned with “official” chemical classifications used in product labeling (Classification, Labelling, and Packaging [CLP] regulation) (EC 2008; UN 2017) or with the substance hazard values used in the EU Ecolabel detergent ingredient database (EC 2016). In other words, what is considered very toxic in CLP and/or the EU Ecolabel, should also have a very toxic indicator for the EU‐EF.

It has been shown that substance toxicity values (used to rank chemicals) or overall product toxicity scores can be very different between these different schemes (Van Hoof etal. 2011; Saouter et al. 2011, 2017a), related to 1) different ecotoxicity data sets and 2) different assessment models with or without different application factors (which are used to derive environmental quality standards from selected data).

In the current USEtox methodology, the freshwater ecotoxicity hazard value of a chemical is based on a compounds’ species sensitivity distribution (SSD) (Fantke etal. 2017). The hazard value represents the hazardous concentration (HC) at which 50% of the tested species are exposed above their chronic EC50. Chronic EC50 data are preferred over acute EC50 data, but an extrapolation factor of 2 for organic chemicals is employed by USEtox to convert acute EC50 to chronic EC50 for chemicals where insufficient or no chronic EC50 aquatic ecotoxicity data are available (Fantke etal. 2017). Three limitations were identified in the approach currently applied by USEtox. The first is that the number of chronic EC50 values is rather limited (chronic test data are usually reporting NOEC, LOEC, or EC*x*), which led to the use of acute toxicity results via an extrapolation factor of 2 for all chemicals. The second limitation is that the large majority of chronic toxicity results available on chemicals expressed as NOEC, LOEC, and EC*x* are not used within USEtox. Finally, the use of a factor of 2 to extrapolate from the acute EC50 to the chronic EC50 values for organic substance may be too simplistic.

The main purposes of the present article are these:

1) Describe the type of ecotoxicity data available in the JRC‐REACH database.
2) Compare different methods to calculate chemical hazardous values using SSDs as recommended in the USEtox approach.
3) Propose new acute‐to‐chronic extrapolation factors specific for algae, crustaceans, and fish.
4) Compare the chemical toxicity ranking (from very toxic to not toxic) that results from different calculation methods against the ranking that results from the EU harmonized classification and labeling, as well with the predicted no‐effect concentration (PNEC) available in the REACH database.

In the *Conclusions and Recommendations* section, the pros and cons for deriving substance hazard values from the REACH database via SSDs is discussed and recommendations are provided.

The present paper does not address nor discuss any aspects of the USEtox model, nor the potential issues associated with the underlying life cycle inventory database to perform a full life cycle assessment (LCA) or EU‐EF study, nor the value or reliability of the CLP or the United Nations’ Globally Harmonized System of Classification and Labelling of Chemicals (GHS) (EC 2008; UN 2017) system, nor the principles behind the derivation of the PNEC used in risk assessment.

## MATERIALS AND METHODS

### JRC‐REACH ecotoxicity database

The European regulation REACH aims to guarantee a high level of human health and environment protection from the risks posed by substances (EC 2006). The selection procedure to extract data from the REACH ecotoxicity database (about 305 068 test results; download date March 2015) is described in Saouter etal. (2019). This extraction procedure was developed to ensure that data met our quality requirements for calculating substance hazard values for use in the EU‐EF. In short, the selection is based on the use of test descriptions, such as duration, endpoint, Klimisch score (Klimisch etal. 1997), and study type, to separate “high‐quality” acute and chronic test data. The selection procedure led to the creation of a new database containing only 54 353 test data points (called the “JRC‐REACH database”), resulting in the exclusion of about 82% of the ecotoxicity data originally recorded in the REACH database (Saouter etal. 2019).

Toxicity data have been pooled into 3 categories: acute EC50_eq_, chronic EC50_eq_, and chronic NOEC_eq_. The subscript “eq” stands for “equivalent,” meaning that for each category several endpoints may be available i.e., EC50_eq_ includes EC50, IC50 (immobilization), and LC50 (lethal); NOEC_eq_ includes NOEC, LOEC, EC10 to EC20, and threshold of toxicological concern (TTC). It should be noted that, although algae EC50 could be considered as a chronic endpoint (algae multiply many times during the 72‐h exposure period), they have been kept in the acute EC50_eq_ “bin” because this is how these data are considered in USEtox and in the EU regulatory schemes. The pooling of endpoints is used to ensure that a toxicity value is available for as many substances as possible. This pooling gives a fair indication of the overall acute and chronic toxicity of substances using all toxicity information available for each specific substance, provided the data meet certain quality criteria (Saouter etal. 2019). Note that this approach is not recommended in the chemical safety assessments utilized in REACH, in view of the much larger data availability reduction that follows from that approach, related to the large tradeoffs of data reduction on the number and “stability” of compound SSDs.

### Calculating substance hazard values

Three different methods to derive final substance hazard values for potential use in life cycle impact assessment (LCIA) were compared:

- USEtox approach using chronic EC50 and acute EC50 divided by 2, to extrapolate from acute EC50 to chronic EC50 (Fantke etal. 2017).
- Using acute EC50_eq_ data only.
- Using chronic NOEC_eq_ data only.

For each method and following the USEtox methodology, the hazard value is derived from a substance‐specific SSD at 50% potential affected fraction (PAF). For the purpose of the present investigation, values at hazardous concentrations HC10 and HC20 of such SSDs are also considered because they represent the levels usually retained for defining a water protection standard. The final derivation of a substance hazard value entails 1) selection of a preferred test endpoint (acute EC50_eq_, chronic EC50_eq_, and chronic NOEC_eq_), 2) the calculation of the geometric means when multiple test results are available for the same species, and 3) calculation of the HC10, HC20, or HC50 values from the derived SSDs. The SSD‐derived hazard values have been calculated using R studio code (Szöcs 2018) for substances having endpoints available for two or more taxonomic groups. However, as shown in Van Zelm etal. (2007), the uncertainty of the final hazard value increases with a low number of input test data, that is, fewer taxonomic groups, and ranges by a factor of 5 to 1000 when 2 or 3 species were available, respectively. When only 1 taxonomic group is available, the hazard value HC50 is not derived from the SSD approach, but is calculated as the antilog of the arithmetic mean of the logarithmic values of species geometric means (in this case, no HC10 and HC20 were calculated).

### Classification, labeling, and packaging regulation

The CLP regulation (EC No 1272/2008) is based on the UN's GHS (EC 2008; UN 2017). The GHS is an internationally harmonized approach to classification and labeling that ensures consistent and appropriate information is reported on all chemicals, whether the chemical is produced for use within a country or exported or imported by a country. The CLP requires manufacturers, importers, or downstream users of substances or mixtures to classify, label, and package their hazardous chemicals appropriately before placing them on the market. All self‐classified and harmonized classifications are available in the Classification and Labelling (C&L) Inventory that is published on the ECHA website (ECHA 2018a). For the present study, only the harmonized classification and labeling for aquatic toxicity with the hazard statement required on the chemical label codes was retained (e.g., H400, H410, H411, H412, and H413) (Table 1).

**Table 1:** Hazard statement codes for aquatic toxicity, simplified

Aquatic hazard classification	Criteria for hazard classification	Hazard statement	Hazard statement code
Acute 1	≤1 mg/L EC50 (short term)	Very toxic to aquatic life	H400
Chronic 1	≤0.1 mg/L NOEC (long term) for substances not rapidly biodegradable	Very toxic to aquatic life with long‐lasting effects	H410
	≤0.01 mg/L NOEC (long term) for substances rapidly biodegradable		
	Chronic data not available but BCF > 500 (or *K*ow > 4)		
Chronic 2	>0.1 to <1 mg/L NOEC (long term) for substances not rapidly biodegradable	Toxic to aquatic life with long‐lasting effects	H411
	>0.01 to <0.1 mg/L EC50 (long term) for substances rapidly biodegradable		
Chronic 3	>10 to 100 mg/L NOEC (long term) for substances rapidly biodegradable	Harmful to aquatic life with long‐lasting effects	H412
Chronic 4	No data to allow for classification, but concern of possible toxicity	May cause long‐lasting harmful effects to aquatic life	H413

The classification and labeling of the ecotoxicity of chemicals was obtained from the European Chemicals Agency (ECHA) from the harmonized classification and labeling table (Table 3.1 of Annex VI to CLP; ECHA 2018b). Chemical hazard values, HC10, HC20, HC50 calculated in the present work, were compared with their official CLP classification. Hazard values (HC50) provided by the USEtox model have also been included in this comparison. By using the CLP toxicity ranking, we are not suggesting that the values used to classify each chemical are particularly more robust and reliable than any other values or schemes used elsewhere. We are using it because there is an international consensus to classify chemicals according to the CLP/GHS approach, and until it is proven to be wrong, a chemical that is classified as very toxic in CLP/GHS should also be classified as very toxic in EU‐EF.

### Predicted no‐effect concentrations

Predicted no‐effect concentrations are used in environmental safety assessment to characterize the concentration below which effects on organisms are deemed acceptable. The PNEC is usually derived from 3 species and from 3 trophic levels: a producer (algae), a first consumer (crustacean), and a carnivore (fish). The lowest ecotoxicity value (LEV) from 3 tested trophic levels is divided by an assessment factor (AF) between 10 to 1000, depending on the number and type of toxicity test available (acute or chronic), to obtain the final PNEC. This approach is similar to the one used in CLP, and it can be used to compare chemical toxicity ranking before and after the safety factor is applied.

The hazard values calculated via the JRC‐REACH database (the present work) were compared with the PNEC values available in the ECHA chemical dissemination website (https://echa.europa.eu/home). The PNEC values in the ECHA dissemination website have been proposed by the registrant of the REACH dossier (usually industry) based on a different curation strategy than the one we have applied. These values were retrieved by Gustavsson etal. (2017) and were made available via their supplementary online material (about 2240 PNEC and AF values). Similarly to the comparison with the CLP ranking, comparing our values with both the REACH‐registered LEV and PNEC is a way to assess whether the toxicity rankings are congruent.

## RESULTS AND DISCUSSION

### Using the JRC‐REACH database

In the JRC‐REACH ecotoxicity database, 54% of the toxicity tests are acute exposures (4853 substances), 40% are chronic expressed as NOEC or ECx (5560 substances), and only 6% are expressed as chronic EC50 (1113 substances) (see Figures S1 and S2 in Supplemental Data). It should be noted that the selection procedure applied to the original REACH database led to the exclusion of 83% of the available ecotoxicity data (not meeting minimum quality requirements). All the selection procedure is described in Saouter etal. (2019). More than two‐thirds of the data are reported for organic substances (see Table TS1 in Supplemental Data), and for the large majority of substances (>95%) only 3, 2, or even 1 taxonomic toxicity data are available per substance (Table 2; Figure S2 in Supplemental Data). The large majority of the tests have been performed on algae, crustaceans, and fish.

**Table 2:** Number of taxonomic groups and substances available for the 3 methods retained to calculate substance hazard values

Method	Nr taxonomic groups	Nr substances	Percent
Acute EC50_eq_	1	1420	29%
	2	1368	28%
	3	1827	38%
	4	123	3%
	5	54	1%
	6	41	1%
	7	1	0%
	8	13	0%
	9	6	0%
Chronic EC50_eq_	1	829	74%
	2	196	18%
	3	28	3%
	4	29	3%
	5	20	2%
	6	10	1%
	7	1	0%
Chronic NOEC_eq_	1	3254	59%
	2	1246	22%
	3	834	15%
	4	46	1%
	5	16	0%
	6	19	0%
	7	119	2%
	8	15	0%
	9	11	0%

eq = equivalent.

From the JRC‐REACH database, selecting only the organic substance (the USEtox domain of application is best fit for organic), 4008, 3777, and 4308 individual organic substance hazard values (HC50) were calculated using the USEtox approach, or using only acute EC50_eq_ and chronic NOEC_eq_, respectively (see TS2, TS3, and TS4 in Supplemental Data).

### Comparing substance hazard values

**Figure 1:**
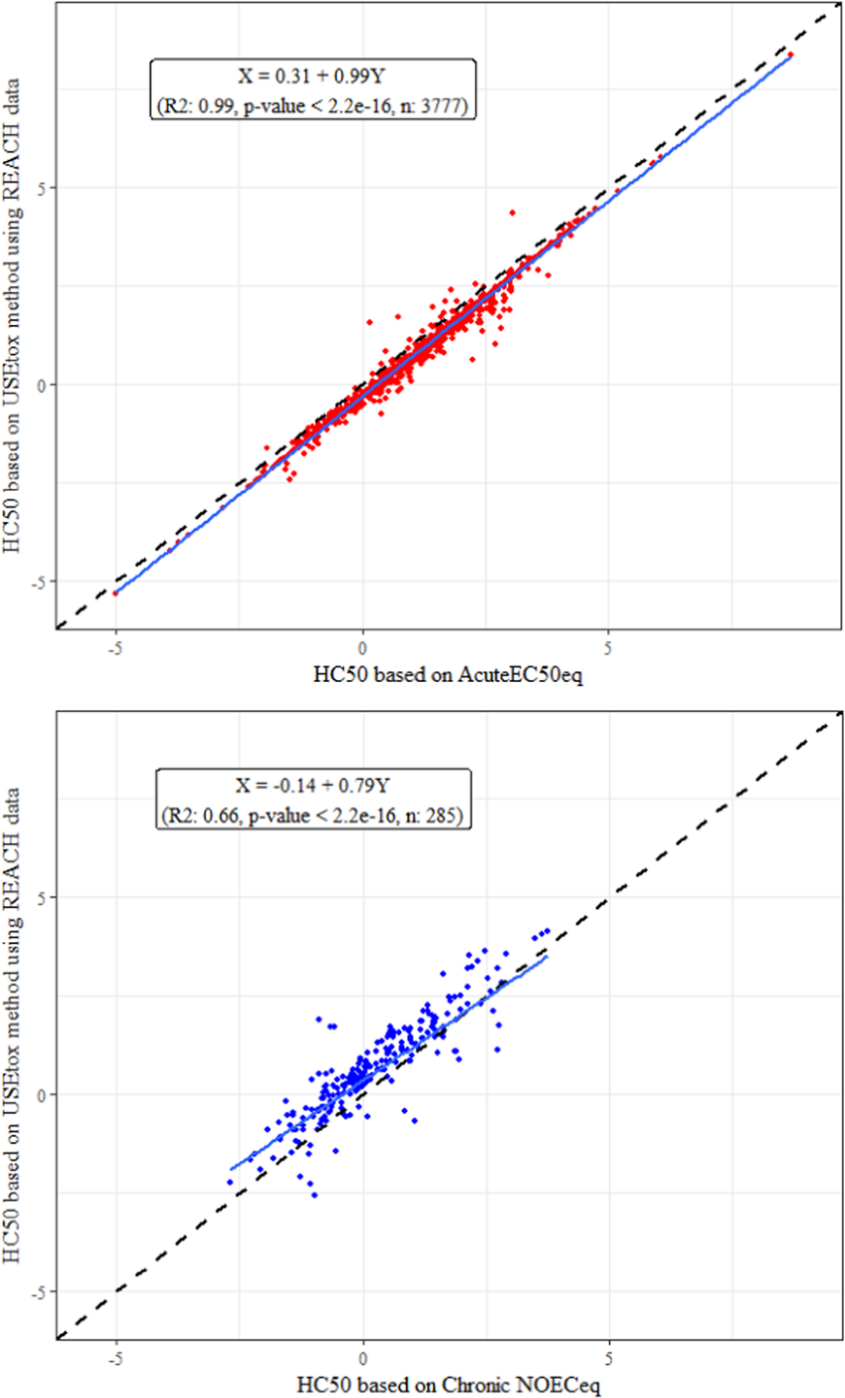
Relations between substance hazard values (HC50) calculated using the USEtox approach and acute EC50_eq_ from the JRC‐REACH data only (top graph) and the chronic NOEC_eq_ (bottom graph). The dashed black diagonal line represents a 1‐to‐1 relation; the blue line represents the best fit regression line. eq = equivalent; HC = hazardous concentration; JRC = Joint Research Centre; NOEC = no observed effect concentration; REACH = European Union Registration, Evaluation, Authorisation and Restriction of Chemicals regulation.

The comparison of the final hazard values based on 50% effect (HC50) showed that applying the USEtox approach on the JRC‐REACH database provided the same outcome as when values are calculated with only acute EC50_eq_ (*R* ^2^ of 0.99; Figure 1, top). This is due to the fact that 92% of the ecotoxicity data in the USEtox approach were derived from acute EC50 values divided by 2. As visible in Figure 1 (top) the USEtox approach provides an overall lower value than when only acute EC50 are used because in the USEtox approach all acute values were divided by 2 (red dots below the dashed dark line representing 1‐to‐1 relation). This observation is probably also valid for the original USEtox database because the number of chronic EC50 values is low (in all databases) and most of the data are based on acute toxicity tests (i.e., EC50). When the comparison is made with HC50 values calculated from chronic test data (i.e., NOEC_eq_), the relation with USEtox HC50 values indicates a larger dispersion (*R* ^2^ of 0.66; Figure 1, bottom) of the data and an underestimation of the chronic values as compared to the current the USEtox approach (Figure 1, bottom). In this case, the USEtox estimations are well above the value calculated with chronic NOEC_eq_ from the JRC‐REACH data, which suggests that the USEtox method is not a sensitive estimate of chronic effect as presented in the USEtox documentation (Fantke etal. 2017) (see blue dots above dashed dark line in Figure 1, bottom). For the comparison of chronic hazard values, only substances having toxicity data from at least 4 species from the JRC‐REACH database were retained (using the most robust estimation).

**Figure 2:**
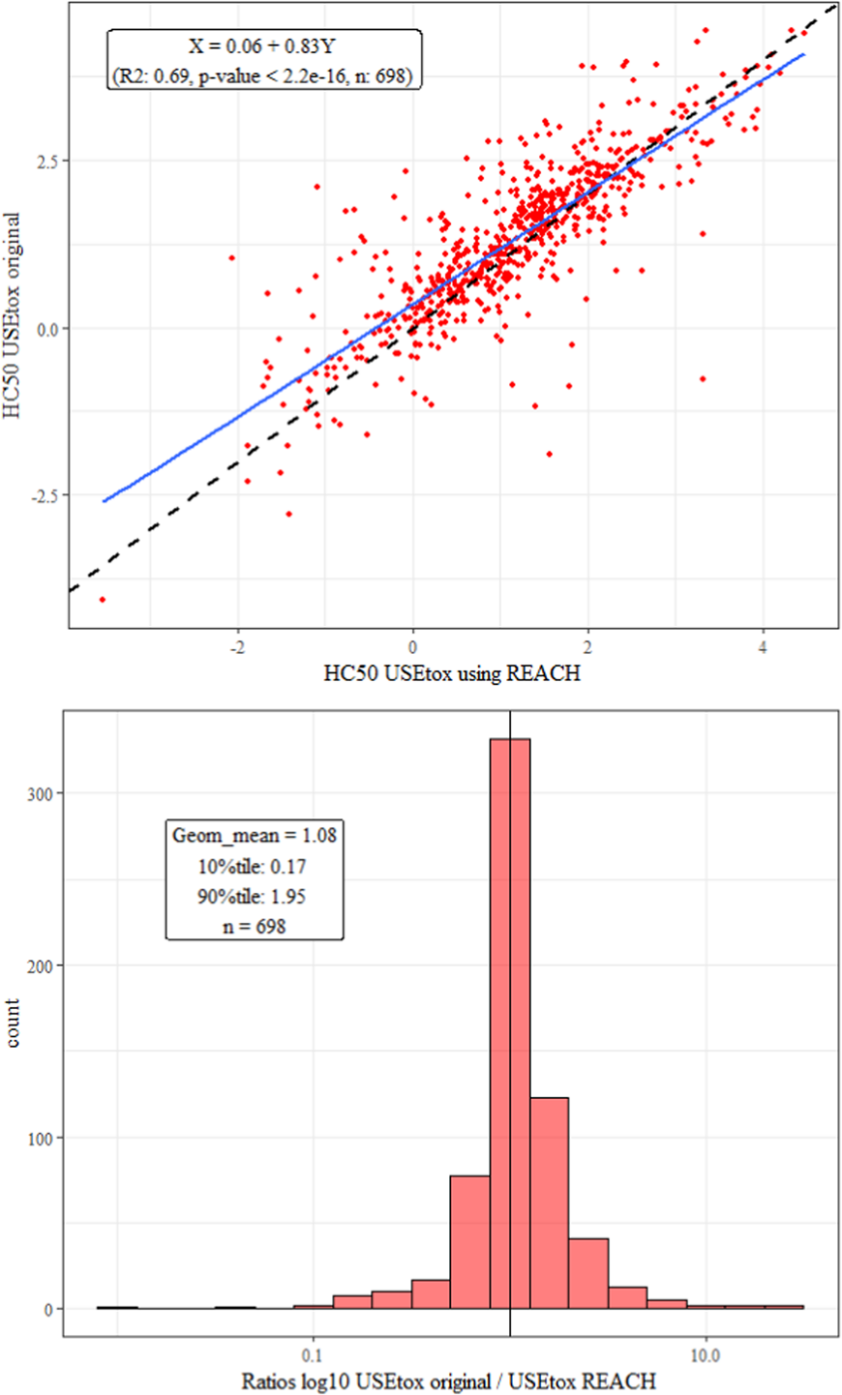
Relations between substance hazard values (HC50) calculated using the USEtox approach with the JRC‐REACH database and the original HC50 values provided with the USEtox model. Scatter plot with the diagonal dashed black line represents a 1‐to‐1 relation and the blue line the best fit (*R* ^2^ = 0.69) (top). The HC50 ratio of USEtox original values and the ones calculated with the JRC‐REACH database (bottom). The vertical line represents the ratios = 1. HC = hazardous concentration; JRC = Joint Research Centre; REACH = European Union Registration, Evaluation, Authorisation and Restriction of Chemicals regulation.

The comparison of the USEtox approach applied on the JRC‐REACH data with the original values provided with the model shows a good fit for most of the data, with few hazard values being orders of magnitude different (Figure 2). However, the ratios show that USEtox hazard values tend to be higher (less toxic) than when the method is applied to the JRC‐REACH database. No investigation has been conducted to explain these differences because the raw data used by the USEtox model are not publicly available. Although the selection procedure of the underlying ecotoxicity data made by the USEtox model and the present work is most likely very different (different original database, different data selection procedure, etc.), the set of outcomes is however robust with respect to the relative ranking of chemicals to pose harm (results of different methods are overall well correlated).

### Acute‐to‐chronic extrapolation factors

**Figure 3:**
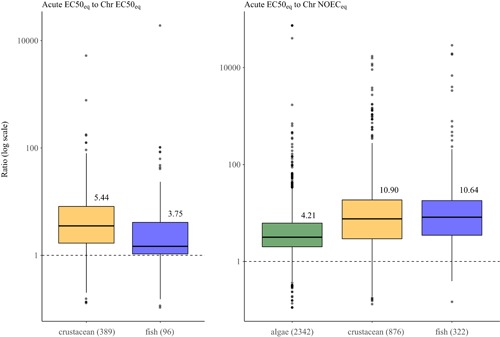
Acute‐to‐chronic ratios for substance having toxicity values on the same species. The black straight horizontal line inside the box represents the median, each upper and lower box represent each a quarter of the database, the black vertical line represents the maximum and minimum of the distribution, and single dots represent individual values (outliers). The value above the box represents the geometric mean (non‐log) and *n* represents the number of species for which the ratio is above 1.

**Figure 4:**
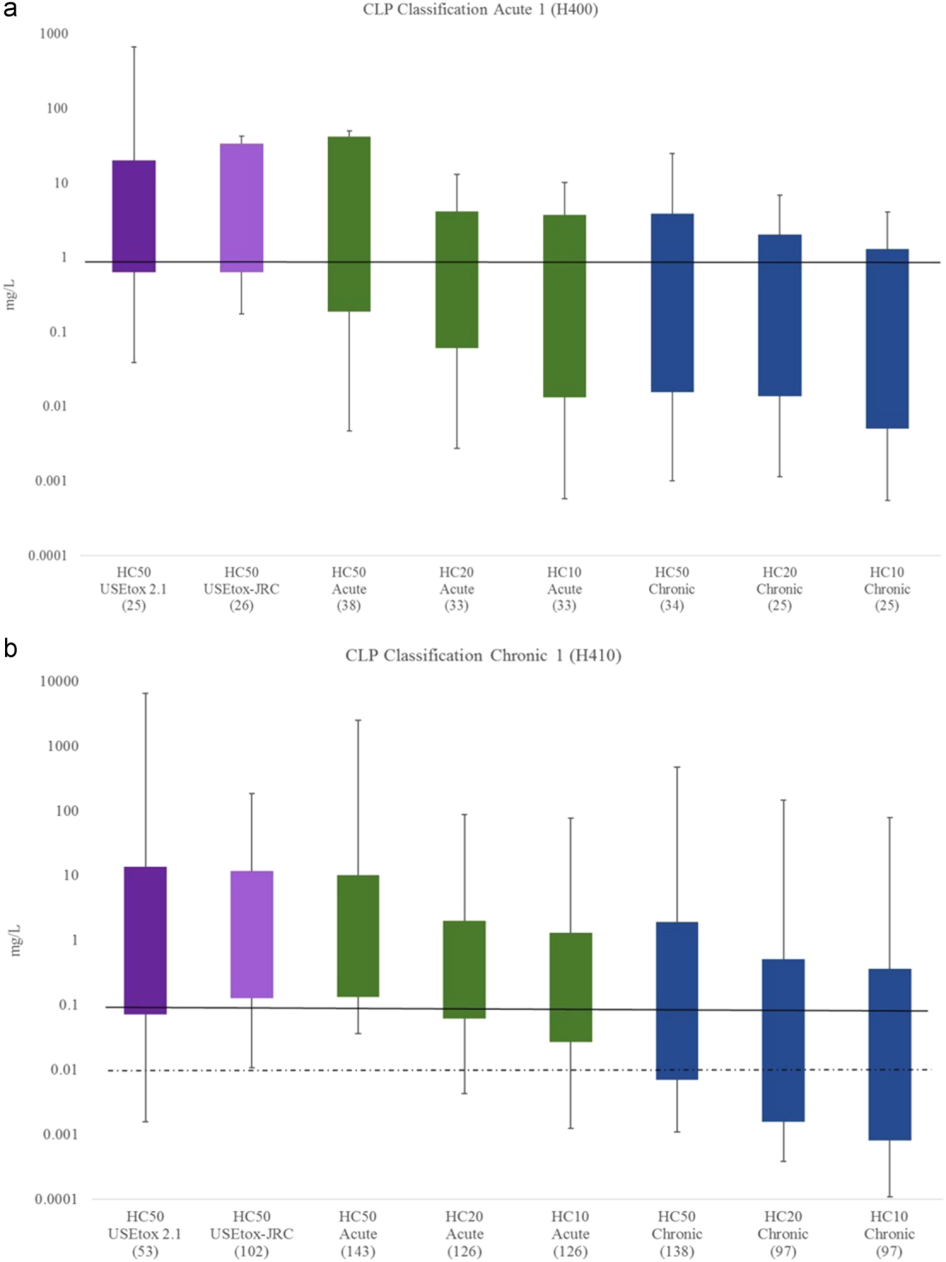
Comparison of the substance harmonized classification with the values obtained with the 3 methods plus original USEtox values with H400 classification (a) and with H410 classification (b). Each box represents 80% of the values. Each vertical line represents the dispersion of the remaining 10% of the values. The solid horizontal line shows the toxicity limit for the H400 (1 mg/L) and H410 CLP classification (0.01 mg/L dashed line and 0.1 mg/L solid line). On the *x*‐axis, the numbers in brackets are the number of chemicals in the JRC‐REACH database that are classified as H400 and H410 according to CLP. CLP = European Union chemical Classification, Labelling, and Packaging regulation; JRC = Joint Research Centre; REACH = European Union Registration, Evaluation, Authorisation and Restriction of Chemicals regulation.

The acute‐to‐chronic relationships calculated for substances having toxicity results for both acute and chronic data for the same species showed a very high spread of the ratios (Figures 3 and 4, Table 3). The statistics presented in Table 3 suggest the extrapolation factor (calculated as geometric mean) for the acute EC50_eq_ to chronic EC50_eq_, is 3.75 and 5.44 for fish and crustaceans, respectively, higher than the factor of 2 proposed by USEtox to be applied to all organic compounds (Figure 3). The extrapolation values have been calculated using only ratios above 1, whereas Figures 3 and 4 show all calculated ratios, including some below 1 (cutoff of 0.1 has been applied to the graph to exclude a few extremely low values). No investigation has been conducted to understand the reason behind ratios below 1, but examination of the full toxicological dossiers may provide an explanation.

**Table 3:** Acute‐to‐chronic ratio for fish, crustaceans, and algae: Acute EC50_eq_ to chronic EC50_eq_ and acute EC50_eq_ to chronic NOEC_eq_ ^a^

ACR	*n*	Minimum	10^th^ percentile	Mean	sdt	Median	Geometric mean	90^th^ percentile	Maximum
Fish acute EC50_eq_ to chronic EC50_eq_ ratios	96	1.01	1.09	208.52	1948.51	2.64	3.74	21.37	19 100
Crustacean acute EC50_eq_ to chronic EC50_eq_ ratios	389	1.01	1.61	25.95	269.74	4.58	5.45	27.14	5263
Fish acute EC50_eq_ to chronic NOEC_eq_ ratios	322	1.03	2.16	276.65	2239.77	8.93	10.64	57.62	28 864
Crustacean acute EC50_eq_ to chronic NOEC_eq_ ratios	876	1.00	2.01	133.02	1037.08	8.77	10.90	78.08	17 245
Algae acute EC50_eq_ to chronic NOEC_eq_ ratios	2342	1.02	1.60	152.24	3155.98	3.40	4.22	14,31	73 729

ACR = acute‐to‐chronic ratio; eq = equivalent; NOEC = no effect concentration; sdt = standard deviation.

^a^ All these values have been calculated by first excluding the ratio <1.

For acute EC50_eq_ to chronic NOEC_eq_, the extrapolation factor (calculated as geometric mean) is 10.64, 10.90, and 4.21 for fish, crustaceans, and algae respectively (Figure 4). As observed previously, the ratios based on per‐taxon derivation of acute‐to‐chronic ratios can vary by orders of magnitude. The ratios for fish and crustaceans are consistent with values proposed in the literature (ECETOC 2003; Azimonti etal. 2015; May etal. 2016; Kienzler etal. 2016, 2017). The ratios for algae (being about half of the ratio for fish and crustaceans, which is similar to the ratio of chronic EC50 to chronic NOEC for fish and crustacean; see Figure 3), however, suggests that the “Algae Acute EC50” should not be considered as an acute endpoint but rather as a chronic one because the algae are going through several full life cycles during the 72‐h exposure period. In the USEtox approach, algae EC50 are considered as acute and are divided by a factor of 2 to convert them into chronic EC50; however, these data should be considered as already being a chronic endpoint (i.e., chronic EC50).

The amplitude of the ratios suggests that applying 1 single extrapolation factor for all organic chemicals (with possibly different factors for fish, crustaceans, and algae) at the level of acute‐to‐chronic test data bears uncertainties, given that the comparisons show the potential for large under‐ or overestimation of the real (but unknown) chronic toxicity values. It is therefore preferable to develop a method to derive substances hazard values to be used in the EU‐EF procedure without or with a limited use of acute‐to‐chronic extrapolated factors. For example, the method using only acute EC50eq or chronic NOECeq could be the basis for deriving final hazard values. They may result in fewer data points per substance, but at least those are then all based on nonextrapolated values. An alternative that was shown to be more robust is to consider the curve shift between SSD‐acute and SSD‐chronic for a compound, as proposed by Posthuma etal. (2018). In this case, the relation should be based on chemicals having at least 4 to 5 species available in both the acute and chronic SSDs, to avoid having the acute‐to‐chronic ratios (ACRs) be calculated on values carrying potentially very high uncertainty (see Van Zelm etal. 2007).

### Hazard values compared to CLP ranking

**Figure 5:**
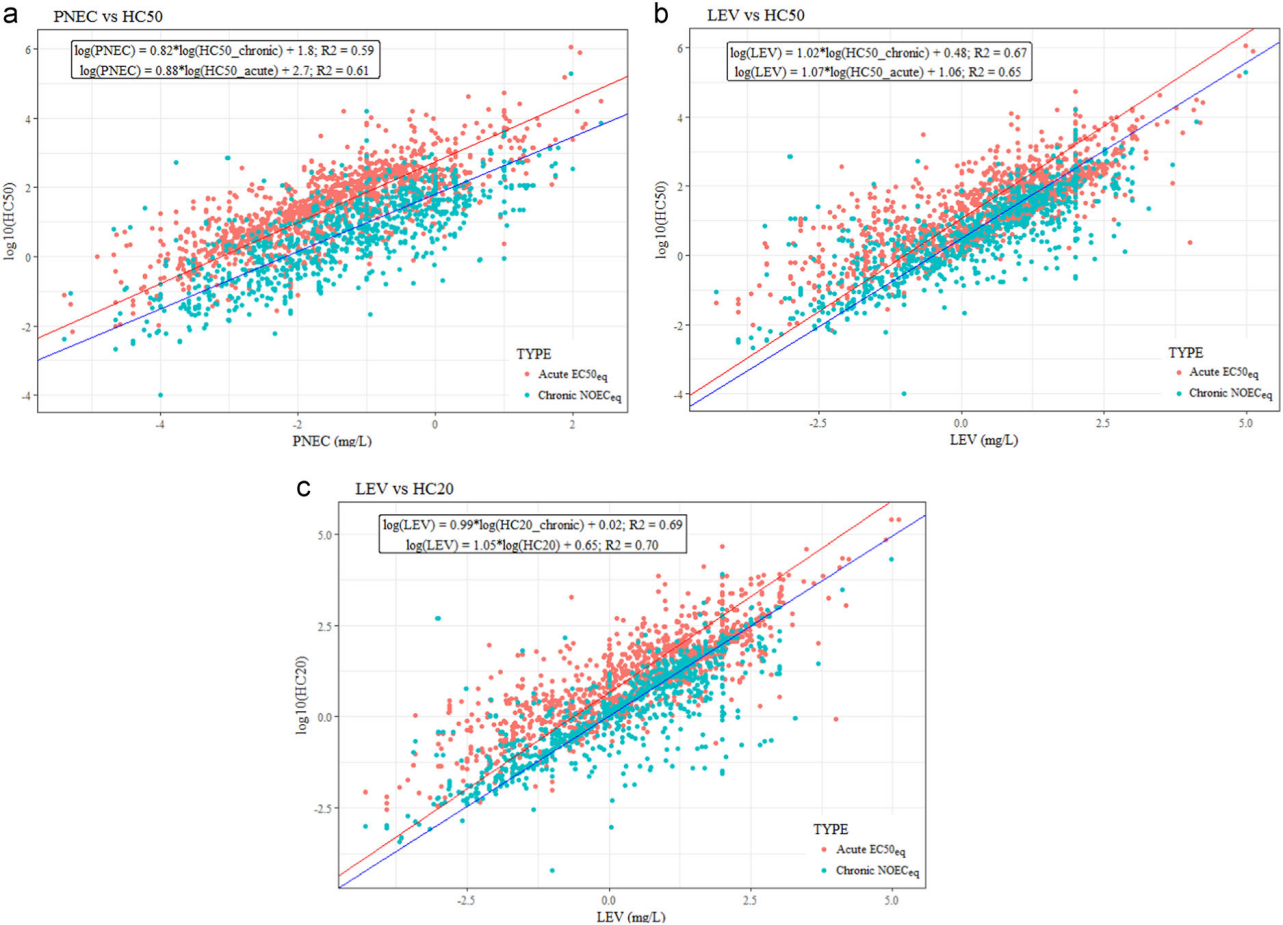
Scatter plots between the PNEC, the LEV used to derive the PNEC, and the HC50 and HC20 derived from acute or chronic SSDs using the JRC‐REACH database. HC = hazardous concentration; JRC = Joint Research Centre; LEV = lowest effect value; PNEC = predicted no effect concentration; REACH = European Union Registration, Evaluation, Authorisation and Restriction of Chemicals regulation; SSD = species sensitivity distribution.

The final chemical hazard values (HC10, HC20, and HC50) calculated using the 3 methods were compared with their corresponding harmonized EU classification and labeling for aquatic toxicity. The HC50 values provided in the USEtox organic database were also included in this comparison. The highest level of classification (H400 and H410) (very toxic to aquatic life and/or having long‐lasting effects) shows that the substances available in the JRC‐REACH database and in the original USEtox database ranked differently when compared to their classification (Figure 5). Most of the values calculated using the USEtox approach (both original values and those calculated with the JRC‐REACH database, using an ACR of 2 as recommended by the original USEtox method) are above the classification threshold for the H400 classification (Acute ≤ 1 mg/L, Table 1). This means that although these substances are considered very toxic to aquatic life according to REACH and CLP (see Table 1), the values calculated by the USEtox method (HC50) suggest they are not. The situation is more balanced when the comparison is made with H410 (Chronic 1 NOEC ≤0.1 mg/L for substances not rapidly biodegradable, and ≤0.01 mg/L for substances rapidly biodegradable). However, in both classifications (H400 and H410), the values calculated with the chronic NOEC_eq_ endpoint show good agreement with the harmonized CLP: Calculated values clearly indicate that the substances are very toxic to aquatic life (H400) or are very toxic to aquatic life and have long‐lasting effects (H410). The same pattern is observed with the next level of classification (H411). However, for the H412 level of classification (Harmful to aquatic life with long‐lasting effects), values calculated using the chronic NOEC values suggest a higher toxicity than the harmonized CLP. This indicates that basing the hazard values on chronic NOEC_eq_ may lead to a slight overestimation of the toxicity of the substance, when they are only considered as harmful according to CLP; however, for substances considered very toxic to toxic, there is a very good match (Figure 5).

### Comparing substance hazard values with PNEC

The relation between the hazard values (HC50) calculated with the REACH database to either the PNEC or the LEV is displayed in Figure 5. The comparison with the PNEC showed a higher dispersion of the data than when the LEV are directly plotted (*R* ^2^ for LEV is slightly higher, due to the data influenced by the AF on the plotted value). However, in both cases, there is an overall positive correlation (*R* ^2^ = 0.67 with hazard value derived from HC50). The overall correlation improves slightly if the HC20 are plotted against PNEC and LEV (*R* ^2^ = 0.7). This positive correlation suggests an overall good alignment between values proposed for product labeling and those used for safety assessment. However, the high variability (up to 4 orders of magnitude) between LEV and hazard values calculated with the JRC‐REACH database clearly indicates that the latter should not be used for environmental safety assessment. This due to the fact that the HC50 and HC20 values intend to represent 50% and 20% effects, whereas the PNEC, LEV, and CLP are based on the most sensitive species.

## CONCLUSIONS AND RECOMMENDATIONS

The main objective of the present work was to demonstrate the feasibility of using the REACH ecotoxicity database to derive toxicity hazard values for a large number of substances in the context of EU‐EF method. As a matter of fact, in the context of a product EU‐EF assessment, if a substance is recorded with an emission to air, water, or soil and does not have any toxicity indicators associated with it (because a value was not yet calculated), the substance is wrongly considered as having no potential impact. Therefore, absence of an ecotoxicity indicator would lead to misinformation on the environmental impacts of a product or service, often wrongly interpreted as a better product environmental profile. To avoid this situation, a priority for comparative product impact assessment was to generate ecotoxicity values for as many substances as possible, avoiding the serious assessment trade‐off that follows from an overly specific selection of test data, which may come at the expense of a potential lowered accuracy for data‐poor substances. Note that Posthuma et al. (2018) assigned SSD‐quality scores to SSDs for this reason, to support assessment design and interpretation, while proving SSDs for 12 386 compounds. It should be recognized, however, that this approach leads to a situation in which, for a significant number of chemicals, the final proposed hazard value to be used in LCA or EU‐EF studies carries a large uncertainty that could seriously impact the interpretation of the final product toxicity score. The uncertainty associated with the derivation of the chemical effect factors impacts the final product score (up to 7 orders of magnitude according to Douziech et al. 2019). Practitioners need to be aware of this trade‐off.

Thanks to the EU REACH legislation, more substances could now be included in the EU‐EF ecotoxicity assessment method for freshwater impacts of chemicals. More than 5550 substances hazard values could be derived (based on chronic NOEC_eq_), compared to the 2499 values available with the USEtox model, or the 239 available in the Detergent Ingredient Database used in the EU Ecolabel (EC 2016). With the further expanded (combined) databases of Posthuma et al. (2018), the number of chemicals that can be evaluated expands to 12 386.

Substance hazard values ranking (from very toxic to non toxic) calculated from the JRC‐REACH database, and using HC20 based on chronic HC*x* values, are in overall good agreement with the official chemical classification and labeling, as well as in line with the values used for safety assessment. However, due to the low number of taxonomic groups available for the large majority of chemicals as the result of the extraction rules applied to the original REACH database (see Table 2), the use of the SSD approach may lead to under‐ and overestimation of chemical toxicity, heavily influenced by potential outliers. Because substance‐specific SSDs are usually derived from a minimum of 8 taxonomic groups in the regulatory context of chemical safety assessments (EC‐JRC 2003), the generation of a substance‐specific SSDs compliant with the chemical safety assessment approach would be limited to a very small portion of the database (less than 1% of the compounds). This limitation would imply that 99% of the compounds would not be considered for the EU‐EF impact assessment, despite the large number of data (representing a serious trade‐off between data selection effects and inability to judge most chemicals). For that reason, and to comply with the USEtox methodology, the SSD approach was still considered despite the high number of substances having fewer than 8 taxonomic groups. Other approaches to derive final substance hazard values, such as lower toxicity value per substance or weighted average, could be considered, but this was out of scope of the present paper (EC 2018b; Saouter et al. 2018).

When used in an EU‐EF context, if a specific substance is found to contribute exceptionally highly to the overall toxicity score, it is recommended to scrutinize the underlying data. The procedure proposed in the present research aims to identify the chemicals that contribute most to the EU‐EF but should not be used directly to limit the use of or to ban chemicals from products.

The database used for the present work has several limitations:

- Despite a selection procedure that differs from the one applied for a regulatory chemical safety assessment (the selection procedure applied in the present work is less strict), the overall lack of ecotoxicity data limits greatly the robustness of the final outcome. Only 40% of the substances in the JRC‐REACH database have 3 or more acute (short‐term) toxicity data and only 13% have 3 or more chronic (long‐term) toxicity data. A large proportion of values have been derived with only 1 species toxicity value, especially if only chronic toxicity endpoint is used (62% have only 1 species toxicity value versus 31% for acute toxicity).
- The selection procedure was based on the criteria applied to evaluate the validity of the information entered by the registrant, which appeared to be of variable quality. There is a clear need for a higher level of standardization of how toxicity tests are recorded in the International Uniform Chemical Information Database (IUCLID).

The second objective of the present work was to test the applicability of the USEtox method to derive substance hazard values and to test 2 new approaches (using only acute data or only chronic EC*x*). The following conclusions are derived:

- The USEtox model only makes use of chronic EC50 values and acute EC50 values divided by 2 (to extrapolate to chronic EC50), leading to final substance toxicity values that represent acute toxicity and not chronic toxicity, as shown in this study. This is due to the fact that chronic EC50 are rare, and most of the database is constituted of acute data. Furthermore, the extrapolation factor of 2 is not appropriate to convert acute EC50 to “real” chronic EC50 for organic substances, as demonstrated in the present work.
- Despite the use of a recent and one of the biggest single‐source ecotoxicity databases, the limited number of species and trophic levels available per substance still considerably limits the possibility to derive robust (i.e., relatively unchanged values when new data would be added) hazard values from SSDs.
- Use of only acute toxicity data generates the same outcome as the USEtox method without the need for an acute‐to‐chronic extrapolation factor. As shown in the present work, applying a unique extrapolation factor at a species‐level acute‐to‐chronic extrapolation, whatever number is chosen, may lead to uncertainties for specific species and compounds and thus to a significant under‐ or overestimation of the “true” chronic toxicity for many chemicals. To avoid adding additional uncertainty in the derivation of the final value, it may be more appropriate to not use an extrapolation factor, or to limit its use as much as possible.
- Using the chronic data expressed as EC*x*, NOEC, and LOEC, which represent the ways chronic toxicity is reported, allows us to calculate “real” chronic substance hazard values; however, a large majority are based on 1 or 2 species toxicity values, given the selected database and data criteria.

A third objective was to compare the different approaches to derive final substance hazard values to the agreed regulatory toxicity ranking of the GHS and CLP. On this point, the main conclusions are these:

- The final hazard values calculated with the JRC‐REACH database that best match the EU harmonized classification are those derived from chronic SSD reference points HC10 and HC20.
- Although the substance hazard values generated with the JRC‐REACH database are not to be used for classification and labeling, nor for safety assessment, achieving overall agreement between the CLP categorization and the EU‐EF makes the interpretation and communication of the results of the EU‐EF more relevant and easy to understand.

It is recommended that the hazard values be derived either from chronic EC*x*, NOEC, and LOEC if the concern is chronic exposure, or from acute EC50, which provides the advantage of greater availability of data. Future substance tests are also more likely to be done on acute than on chronic exposure. It is also strongly recommended to make use of all the existing ecotoxicological databases to increase the number of species toxicity results per substance, allowing a better use of SSD and consequently reducing uncertainty.

## Disclaimer

The authors declare no conflicts of interest.

## Data Accessibility

All ecotoxicity data have been provided by the European Chemical Agency (ECHA) and are available on the ECHA dissemination website (https://echa.europa.eu/home).

## SUPPLEMENTAL DATA

**Figure S1.** Endpoints reported in the JRC‐REACH ecotoxicity database based on the REACH data.

**Figure S2.** Number of distinct taxonomic groups per number of substances.

**Table S1.** Number of tests per type of substance

**Table S2.** Hazard values calculated according to the USEtox approach

**Table S3.** Hazard values calculated using acute EC50_eq_ only

**Table S4.** Hazard values calculated using chronic NOEC_eq_ only

## Supporting information

This article contains online‐only Supplemental Data.

This article contains online‐only Supplemental Data.
